# RAN Nucleo-Cytoplasmic Transport and Mitotic Spindle Assembly Partners XPO7 and TPX2 Are New Prognostic Biomarkers in Serous Epithelial Ovarian Cancer

**DOI:** 10.1371/journal.pone.0091000

**Published:** 2014-03-13

**Authors:** Katia Y. Cáceres-Gorriti, Euridice Carmona, Véronique Barrès, Kurosh Rahimi, Isabelle J. Létourneau, Patricia N. Tonin, Diane Provencher, Anne-Marie Mes-Masson

**Affiliations:** 1 Centre de Recherche du Centre Hospitalier de l’Université de Montréal (CRCHUM), Montreal, Canada; 2 Institut du Cancer de Montréal, Montreal, Canada; 3 Department of Pathology, Centre Hospitalier de l’Université de Montréal (CHUM), Montreal, Canada; 4 The Research Institute of the McGill University Health Centre, Montreal, Canada; 5 Department of Human Genetics, McGill University, Montreal, Canada; 6 Department of Medicine, McGill University, Montreal, Canada; 7 Department of Obstetric-Gynecology, Université de Montréal, Montreal, Canada; 8 Department of Medicine, Université de Montréal, Montreal, Canada; University of Quebec at Trois-Rivieres, Canada

## Abstract

**Purpose:**

Epithelial ovarian cancer has the highest mortality rate of all gynecological malignancies. We have shown that high RAN expression strongly correlates with high-grade and poor patient survival in epithelial ovarian cancer. However, as RAN is a small GTPase involved in two main biological functions, nucleo-cytoplasmic transport and mitosis, it is still unknown which of these functions associate with poor prognosis.

**Methods:**

To examine the biomarker value of RAN network components in serous epithelial ovarian cancer, protein expression of six specific RAN partners was analyzed by immunohistochemistry using a tissue microarray representing 143 patients associated with clinical parameters. The RAN GDP/GTP cycle was evaluated by the expression of RANBP1 and RCC1, the mitotic function by TPX2 and IMPβ, and the nucleo-cytoplasmic trafficking function by XPO7, XPOT and IMPβ.

**Results:**

Based on Kaplan-Meier analyses, RAN, cytoplasmic XPO7 and TPX2 were significantly associated with poor overall patient survival, and RAN and TPX2 were associated with lower disease free survival in patients with high-grade serous carcinoma. Cox regression analysis revealed that RAN and TPX2 expression were independent prognostic factors for both overall and disease free survival, and that cytoplasmic XPO7 expression was a prognostic factor for overall patient survival.

**Conclusions:**

In this systematic study, we show that RAN and two protein partners involved in its nucleo-cytoplasmic and mitotic functions (XPO7 and TPX2, respectively) can be used as biomarkers to stratify patients based on prognosis. In particular, we reported for the first time the clinical relevance of the exportin XPO7 and showed that TPX2 expression had the strongest prognostic value. These findings suggest that protein partners in each of RAN’s functions can discriminate between different outcomes in high-grade serous epithelial ovarian cancer patients. Furthermore, these proteins point to cellular processes that may ultimately be targeted to improve the survival in serous epithelial ovarian cancer.

## Introduction

Epithelial ovarian cancer (EOC) is the most lethal of all gynecologic malignancies in North America [1] and worldwide. This is attributed to the asymptomatic nature of the disease implying a late diagnosis with a five-year survival rate at 30% [2], [3]. Over the past 30 years, advances in surgery and chemotherapy have had little impact on overall patient survival [4], [5] and current treatment leads to relapse in the majority of the patients. Approximately 80% of EOC patients presents a serous histotype [6], [7] which is categorized according to tumor grade and to clinical stage, representing the degree of cellular differentiation and the spread of the disease [8] respectively. Molecular evidence supports a classification that separates patients with these serous carcinomas in two types: patients with low-grade tumors (LG, well differentiated) and with high-grade tumors (HG, poorly differentiated) [9], [10]. Patients with LG serous tumors typically have a good prognosis but account for 5% of all serous EOCs. Patients with HG serous carcinoma have a poor prognosis with survival at five-years of less than 40% [11]. Research into these two distinct diseases, LG and HG serous EOC, would thus provide a better understanding of ovarian cancer biology and help improve clinical outcomes. Moreover, biomarker discovery discriminating HG serous EOC patients having good or poor prognosis may contribute to patient therapeutic stratification and may increase overall survival.

In previous studies, we have demonstrated that RAN (RAs-related Nuclear protein), in EOC, is over expressed as tumor grade increases and is strongly associated with poor patient survival [12], [13]. Therefore, RAN functions may be deregulated in ovarian carcinomas and RAN expression patterns may be used as a prognostic tool in patients with advanced EOC. *In vitro*, the use of a short hairpin RNA (shRNA) targeting RAN expression prevents EOC cell proliferation, suggesting RAN as a potential amenable therapeutic target for the treatment of this disease [14]. Furthermore, decreasing RAN expression in *in vivo* mouse xenograft experiments resulted in the arrest of EOC tumor growth [14]. These observations indicate that RAN is involved in ovarian cancer progression and might be implicated in tumorigenesis and/or cell survival. These findings correlate well with similar studies in different types of cancer [15]–[19].

At the cellular level, RAN performs two major and distinct functions. At interphase, RAN regulates nucleo-cytoplasmic transport of molecules through the nuclear pore complex [20], [21]. At mitosis, RAN performs a different function and controls cell cycle progression through the regulation of mitotic spindle formation [22].

The RAN-GTP cycle is regulated by three proteins; RCC1, RAN-GAP1, and RANBP1 [23], [24]. RCC1 exchanges GDP for GTP, converting RAN-GDP to RAN-GTP [23]. In contrast, RANBP1 and RAN-GAP1 work to increase GTP hydrolysis [24] and thereby replenish the RAN-GDP pool [25], [26]. RAN uses the same GTP/GDP cycle to regulate both of its physiological functions. However, the gradient GTP/GDP achieved by these regulators is unique to each function of RAN. For nuclear transport, the gradient is established across the nuclear membrane by asymmetric distribution of the regulator proteins [27]. During mitosis, although no membrane separates the regulators, a gradient is achieved in proximity to the chromosome with RCC1 being attached to chromosomes [27].

Although RAN uses the same regulators for GTP/GDP exchange, it utilizes unique partner proteins during interphase and mitosis to perform different physiological functions (transport vs. spindle assembly). During interphase, the enrichment of RAN-GTP in the nucleus is coupled to exportins such as exportin-t (XPOT) [28], [29] and exportin-7 (XPO7 also termed RANBP16) [30], [31], which serve as adaptors by attaching tRNAs and proteins, respectively, to allow nuclear export of their cargos. In contrast, RAN-GDP in the cytoplasm binds to importins, such as importin β (IMPβ) [32], [33], which recognize the nuclear localization sequence (NLS) of proteins to facilitate their movement through the nuclear envelope. During mitosis, RAN-GTP promotes spindle assembly in a manner that is independent of nuclear transport [34]. IMP β binds and inhibits spindle assembly factors, such as TPX2 (Targeting Protein for Xklp2). In proximity to chromosomes, TPX2 is subsequently released facilitating the regulation of microtubule organization and dynamics [35].

The objective of the present study was to investigate which function of RAN would be more relevantly implicated in HG serous EOC malignancy and poor patient survival. To verify the impact of one function *versus* the other, the expression of molecular partners, specific to nucleo-cytoplasmic transport and mitosis, were analyzed by immunohistochemistry on a tissue microarray of 143 serous carcinomas comprising 131 cases of HG and 12 of LG tumors. We evaluated the expression of individual proteins in correlation with clinical parameters and determined their association with EOC progression and outcome. Promising results were obtained for two RAN partners, XPO7 and TPX2, with potential to be clinically relevant in stratifying HG serous EOC patients.

## Methods

### Ethics Statement

The CHUM institutional ethics committee (Comité d’éthique de la recherche du Centre hospitalier de l’Université de Montréal) approved this study and written consent was obtained from patients prior to sample collection.

### Patients and Tissue Specimens

Tumor samples were obtained from patients who underwent surgery for ovarian cancer at the Department of Gynecologic Oncology - Centre hospitalier de l’Université de Montréal (CHUM). A gynecologist-oncologist determined disease stage as defined by the Federation International of Gynecology and Obstetrics (FIGO). An independent pathologist reviewed histopathology and tumor grade. Tissue selection criteria for this study were based on independent confirmation of serous histopathology in samples from chemotherapy-naïve patients. Samples were collected between 1990 and 2006. Patient overall survival was defined as the time from surgery to death from ovarian cancer or last follow-up. Patient disease free survival was calculated from the time of surgery until the first progression. Therefore only patients that progressed were included in our analysis. Clinical data on progression-free interval were defined according to level of blood CA125 and tumor size assessed by imaging. Patients known to be still alive at time of analysis were censored at time of their last follow-up. The ages of diagnosis of patients with LG tumors ranged from 27 to 71 years (average = 48.5 years) and they were followed 50.6 months on average. The ages of patients with HG tumors ranged from 34 to 87 years (average = 62.6 years) and their average follow-up was 37.2 months. The cohort is described in Table S1.

### Epithelial Serous Ovarian Tumor Tissue Microarray (TMA)

All cases were reviewed by a gynecologic pathologist. The grade and type of ovarian carcinoma [9], [10] were identified and areas of interest were marked on slides. Two cores of 1mm for each tissue sample were arrayed onto two recipient paraffin blocks. This tissue array was composed of cores from 12 LG and 131 HG tumors (2 cores per patient sample) of the serous histopathological subtype and six paraffin-embedded EOC cell line pellets as staining controls (292 total cores). The TMA was then sectioned, stained with hematoxylin-eosin and subjected to another review of tissue pathology to verify the presence of tumor material in each core.

### Western Blot Analysis

The quality and specificity of individual antibodies where verified by Western blot analysis. Total protein extracts (30 µg) were loaded and electrophoresed on SDS-polyacrylamide gel then transferred onto a nitrocellulose membrane. The membranes were blocked with 5% milk-PBS (1 hr) and subsequently probed with primary antibodies (2 hrs at room temperature) at the optimal dilutions (1∶500 for RAN, XPO7, XPOT and TPX2; 1∶200 for IMPβ; 1∶300 for RCC1; 1∶75 for RANBP1). Each primary antibody was detected with an HRP-conjugated secondary antibody (Santa Cruz Biotechnology Inc.) and visualized by the enhanced chemiluminescence (ECL) method (GE Healthcare, UK). Beta-actin was used as a loading control (1∶50,000) (Abcam, Cambridge, MA).

### Immunohistochemistry (IHC)

For the manual staining method, the TMA block was sectioned at 4 µm on superfrost+ glass microscope slides (Fisher Scientific Limited, Nepean, ON, Canada). Slides were heated at 60°C for 15 min, deparaffinized in xylene, rehydrated in an ethanol gradient, and washed in phosphate-buffered saline (PBS). To unmask antigens, slides were placed for 20 min at high temperature under high pressure in citrate buffer (10 mM sodium citrate, 0.05% Tween 20, pH 6.0) for staining with anti-RCC1 (1/50, SC-1161, Santa Cruz Biotechnology Inc.), anti-IMPβ (1/25, SC-1863, Santa Cruz Biotechnology Inc.) and anti-TPX2 (1/100, NBP1-01041, Novus Biologicals), or in citrate-EDTA buffer (10 mM sodium citrate, 1 mM EDTA, 0.05% Tween 20, pH 8.0) for staining with anti-RANBP1 (1/25, SC-1159, Santa Cruz Biotechnology Inc.), anti-XPOT (1/50, LS-C80366, Lifespan Biosciences Inc.) and anti-XPO7 (1/200, LS-C55360, Lifespan Biosciences Inc.) antibodies. Slides were then incubated with 3% hydrogen peroxide in PBS (to block endogenous peroxidase), and washed in PBS. Tissue sections were blocked with a non-serum protein-blocking reagent (DakoCytomation Inc., Mississauga, ON, Canada) for 15 min at room temperature and incubated with primary antibody for 60 min at room temperature in a humid chamber. Substitution of the primary antibody with PBS served as a negative control. The slides were then washed in PBS, incubated with secondary antibodies conjugated with horseradish peroxidase (Santa Cruz Biotechnology Inc., Santa Cruz, CA). Reaction products were developed using diaminobenzidine (DAB) containing 0.3% hydrogen peroxide up to 5 min. Cell nuclei were counterstained with diluted hematoxylin for 1 min.

For the automated staining method, sections of formalin fixed paraffin embedded tumors (4 µm) were stained using the BenchMark XT automated stainer (Ventana Medical System Inc., Tucson, AZ). Antigen retrieval for RAN was performed with Cell Conditioning 1 (Ventana Medical System Inc.) for 30 min. Slides were incubated with anti-RAN antibody (1/100, SC-1156, Santa Cruz Biotechnology Inc.) for 40 min, and developed by the iView DAB detection kit (Ventana Medical System Inc.). Hematoxylin and bluing reagent were used for counterstaining (Ventana Medical System Inc.). TMAs were observed by brightfield microscopy and digitally imaged (Aperio ScanScope, Vista, California, USA).

### Staining Quantification

Protein expression by IHC was scored according to the subcellular localization and staining intensity of malignant cells. Nuclear and cytoplasmic expression of RAN network proteins in ovarian cancer tissues was observed using digitally imaged scans of each stained TMA and scored according to the intensity of staining. For each RAN network protein in epithelial zones of the tumor cores, the staining intensity of DAB was defined as 0 (no staining), 1 (weak staining), 2 (moderate staining) and 3 (strong staining). All TMAs were analyzed in a blind study by two independent observers. Inter-rating correlation (ICC) was greater than 75% for all assays. The average of all cores with cancer from the same patient was used for analysis. When strong differences in scoring between the two observers occurred the core was re-evaluated to reach a consensus between the two observers.

### Statistical Analysis

All statistical analyses were performed using SPSS software version 16.0 (SPSS Inc., Chicago, IL, USA) where p<0.05 were deemed significant. Protein expression patterns of RAN partners were correlated (Pearson correlation test, two-tailed) with the protein expression of RAN and to disease stage (I to IV). Survival curves (overall survival and disease free survival) were plotted by the Kaplan-Meier estimator, compared using the log rank test and analyzed for significant differences. For each RAN partner, the number of patients in every survival curve is reviewed in Table S2. Univariate and multivariate Cox proportional hazard models were used to determine the hazard ratio for each marker. Multivariate analyses were done using a hazard model with an enter method. A maximum of four independent variables were included in the multivariate Cox regression model to avoid over-fitting.

## Results

### Expression of RAN and its Network Partner Proteins in Serous EOC Tissues

**Figure 1:**
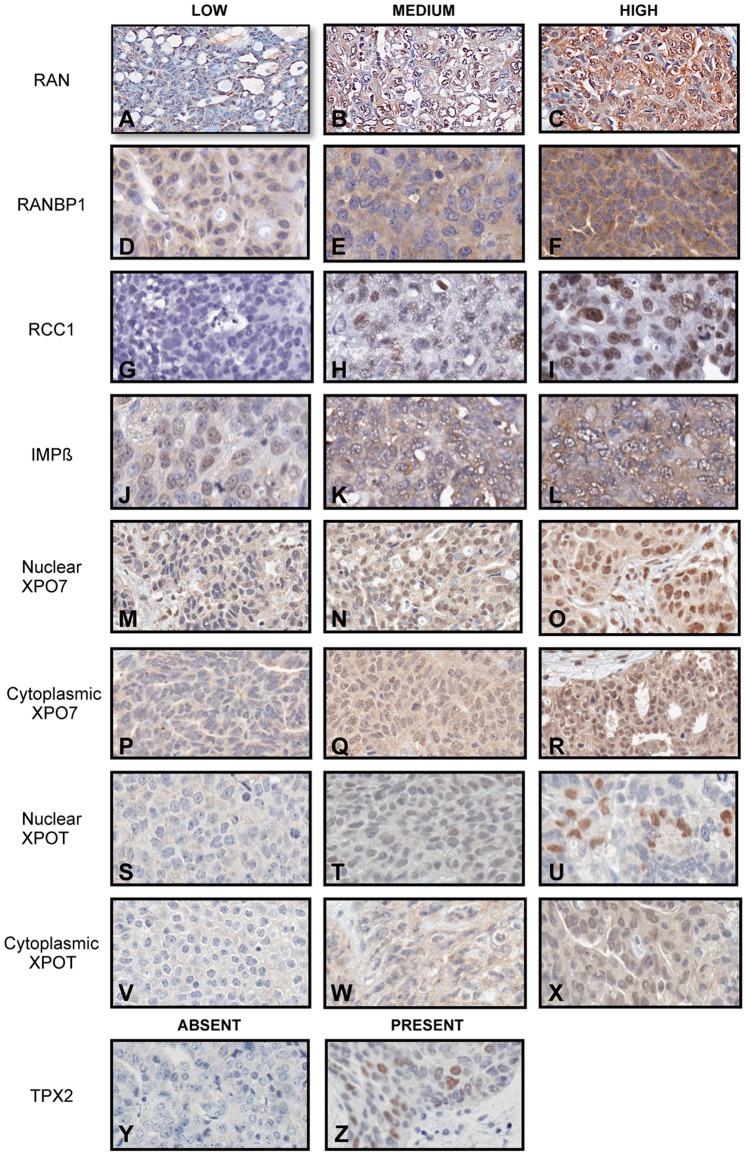
Expression pattern of RAN network proteins in serous epithelial ovarian carcinoma. Representative images of immunoperoxydase staining pattern on a TMA core are shown for each protein and tumor grade (magnification 20×). Quantification of RAN (A–B), RANBP1 (C–D) and IMPβ (G–H) was exclusively for cytoplasmic staining. RCC1 (E–F) and TPX2 (M–N) were exclusively localized to the nucleus. XPO7 (I–J) and XPOT (K–L) were quantified for both their nuclear and cytoplasmic expression.

**Figure 2:**
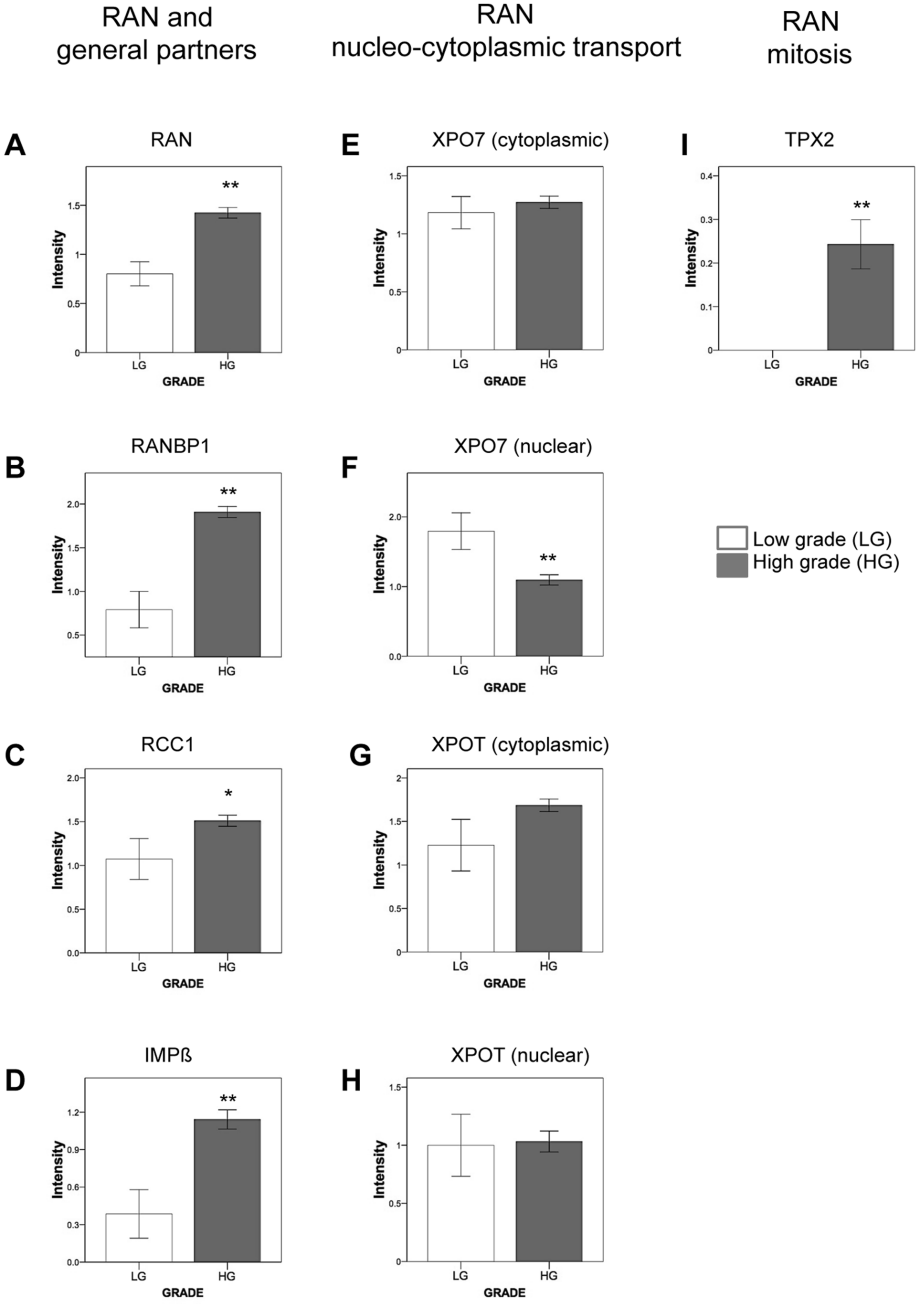
Intensity of RAN network proteins in low-grade and high-grade serous epithelial ovarian cancer. For each protein, the staining intensity was defined as 0 (no staining), 1 (weak staining), 2 (moderate staining) or 3 (dark staining) within the epithelial compartment by two independent observers. The mean of the staining intensity for each protein was compared between low grade (white bars) and high grade tumors (grey bars) using a Student test. *p<0.05 **p≤0.005.

To determine if any of the partners of RAN are implicated in the biology of EOC, we performed an IHC analysis using a TMA containing a total of 286 cores of cancer specimens representing 143 patients. RAN and the six RAN-partner proteins (RANBP1, RCC1, IMPβ, XPO7, XPOT and TPX2), were analyzed using this tissue array. Specificity for each antibody was first evaluated by western blot analysis using cellular extracts of four EOC cell lines (Figure S1) and by IHC using paraffin-embedded cell pellets from the same cell lines (Figure S2). The intensity and localization of the staining were then assessed using digitally imaged scans of each stained TMA. Nuclear and cytoplasmic expression of RAN network proteins in EOC tissues were observed and classified according to the average intensity of staining as low (scores <1), medium (scores = 1<2) and high (scores ≥2) (Figure 1), except for TPX2, which was classified as absent (scores = 0) or present (scores >0), because of the unique staining pattern of this protein (Figures 1–2).

The expression of RANBP1 (GDP) and RCC1 (GTP), which are general proteins related to the RAN-GTP cycle, were initially investigated. IMPβ expression was analyzed to concomitantly evaluate both of the two main functions of RAN. Then we specifically explored the nucleo-cytoplasmic function of RAN by assessing the expression of two exportins: XPO7 and XPOT. Finally, we examined TPX2 expression in order to evaluate the spindle assembly during mitosis, another important function of RAN. Figure 1 shows representative images for the staining of each protein in HG serous ovarian carcinomas. Expression of RAN (Figure 1A–C), RANBP1 (Figure 1D–F) and IMPβ (Figure 1J–L) were localized to both the cytoplasm and the nucleus, although signal quantification focused exclusively on the cytoplasm based on its higher expression in this cellular compartment. Expression of RCC1 (Figure 1G–I) and TPX2 (Figure 1Y–Z) were nuclear. Nuclear and cytoplasmic staining was observed for XPO7 (Figure 1M–R) and XPOT (Figure 1S–X) proteins, and both were quantified.

### RAN Network Proteins are Correlated with Tumor Grade

Having established the staining pattern for each of the RAN network proteins, we then assessed the staining intensity according to tumor grade (Figure 2). In a previous study, we reported the observation that RAN staining was positively associated with increased tumor grade in serous EOC [13]. In the present study, using an enriched patient cohort, we further corroborate our initial finding showing that levels of cytoplasmic RAN were significantly higher in HG tumors compared to LG tumors (p<0.001) (Figure 2A). The cytoplasmic staining intensity of RANBP1 (p<0.001), IMPβ (p = 0.048), and nuclear localization of RCC1 (p = 0.004) were significantly higher in HG versus LG tumors (Figure 2B–D). For the nucleo-cytoplasmic RAN network proteins, nuclear XPO7 was significantly less expressed in HG than LG tumors (p = 0.005), whereas no significant differences were observed for cytoplasmic XPO7 staining or for cytoplasmic/nuclear XPOT staining (Figure 2E-2H). Interestingly, unlike with all other RAN network proteins investigated, staining for the mitotic RAN partner TPX2 was found exclusively within the nuclei of HG serous carcinomas (p<0.001) (Figure 2I). Its expression was dichotomized as either positive or absent due to the unique staining pattern of this protein in tumors of patients. Our results suggest that deregulation of the RAN network is characteristic of HG serous EOC, and thus is in line with the recent notion that HG and LG serous EOC are distinct disease entities [9], [10].

We next verified the association between the expression of RAN and each of the RAN network partners using the Pearson correlation test (two-tailed) (Table 1). As expected, the proteins involved in GTP-cycle and IMPβ, were positively correlated with RAN (RANBP1, p<0.001; RCC1, p = 0.026; IMPβ, p<0.001), suggesting a competent RAN GTPase cycle (Table 1). For the nucleo-cytoplasmic role of RAN, a significant positive correlation was found for cytoplasmic XPO7 (p = 0.028) as well as nuclear XPOT expression (p = 0.050) (Table 1). However, no correlation was observed between the staining intensities of RAN and TPX2, which could be explained by the indirect nature of their interaction in mitosis [35].

**Table 1:** Pearson correlation coefficient between the expression of RAN and partners with disease stage or RAN expression itself in EOC tissues.

			Stage	RAN
RAN and	RAN	r*	0.064	1
general		p-value**	0. 466	
partners	RANBP1	r	−0.181	0.326
		p-value	**0.034**	**0.0001**
	RCC1	r	0.023	0.195
		p-value	0.789	**0.026**
	IMPORTIN β	r	0.009	0.302
		p-value	0.919	**0.0004**
Nucleo-	XPO7	r	−0.133	0.205
cytoplasmic	cytopasmic	p-value	0.152	**0.028**
transport	XPO7	r	0.082	−0.044
	nuclear	p-value	0.382	0.644
	XPOT	r	−0.041	−0.019
	cytoplasmic	p-value	0.665	0.845
	XPOT	r	0.081	0.197
	nuclear	p-value	0.422	**0.05**
Mitosis	TPX2	r	0.081	−0.043
		p-value	0.422	0.651

*Pearson correlation coefficient.

**Bold fonts denote significant values at p<0.05.

The association between the expression of RAN or any of the RAN network partners with disease stage was also investigated using the Pearson correlation test (two-tailed) (Table 1). A significant correlation was observed only for RANBP1 staining (p = 0.034), showing a slight negative association (Pearson coefficient = −0.181) (Table 1).

### RAN, XPO7 and TPX2 are Associated with Poor Patient Survival

**Figure 3:**
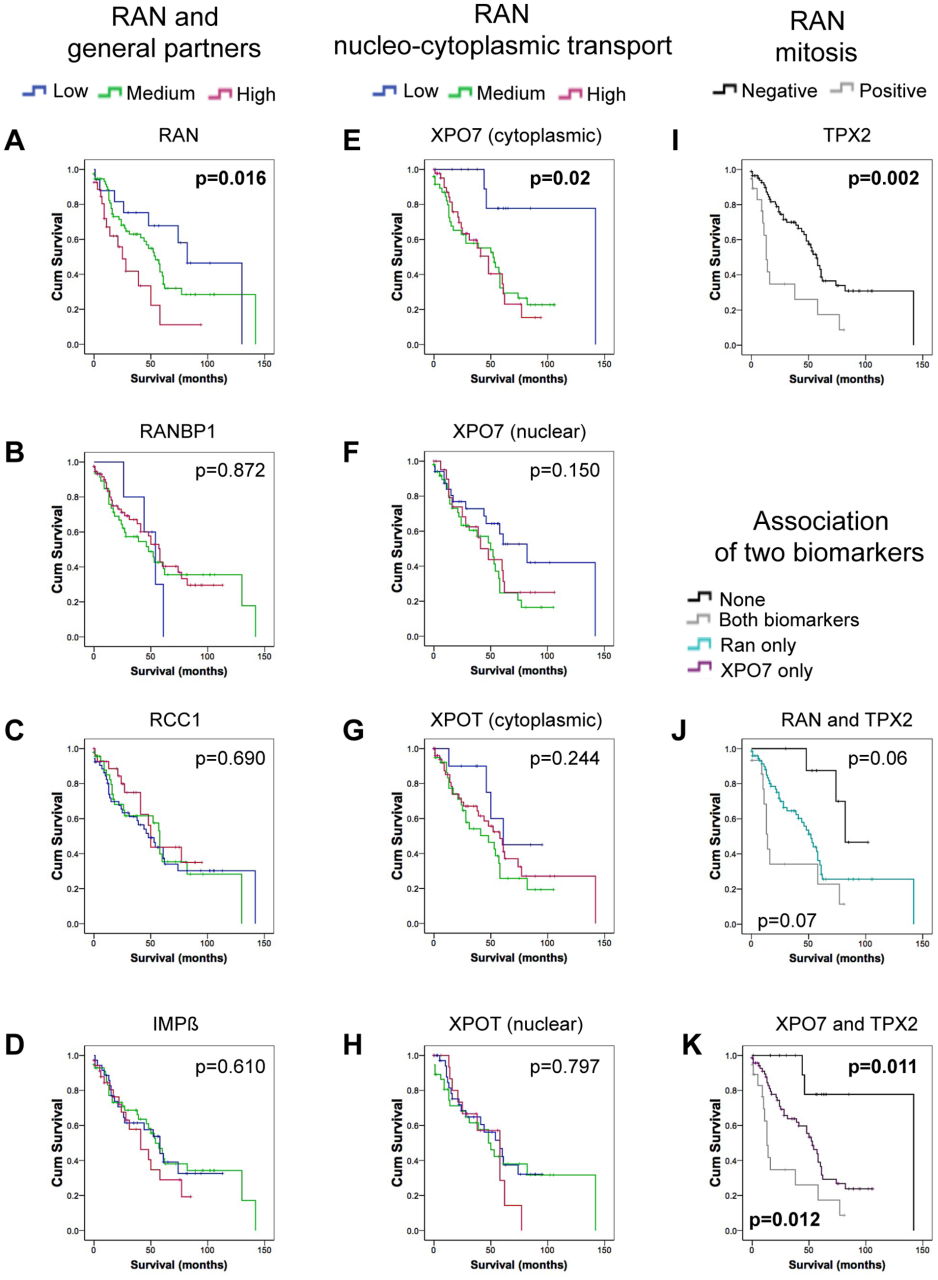
RAN, XPO7 and TPX2 expressions are associated with poor survival in high-grade serous epithelial ovarian cancer. Expression of RAN network proteins was evaluated as prognostic indicators in only the high-grade serous carcinomas by Kaplan–Meier analysis. Survival was defined as the time from surgery to death from ovarian cancer or last follow-up. The log-rank test was used to define statistical difference between groups. For panels A–H, low staining (blue line) defined by values <1, medium staining (green line) defined by = 1<2 and high staining (pink line) defined as ≥2. For panel I, samples are scored as negative (black line) or positive (grey line) for TPX2 staining. In A–I, p denotes significance among all groups. For panels J–K, negative TPX2 staining with low staining intensity of RAN (J –black line) or XPO7 (K – black line); negative TPX2 staining with medium+high staining of RAN (J – cyan line) or XPO7 (K – violet line); positive TPX2 staining with medium+high staining of RAN (J – grey line) or XPO7 (K – grey line). In J–K, p (top right hand corner) is calculated for black versus cyan (RAN, panel J) or violet (XPO7, panel K). In J–K, p (bottom left hand corner) is calculated for grey versus cyan (RAN, panel J) or violet (XPO7, panel K). A–K, p<0.05 are indicated in bold letters.

We investigated the relationship between the expression of RAN partner proteins and overall survival in the cohort of 143 patients. Kaplan–Meier survival curves were derived and compared by the log-rank test for HG cases only (Figure 3, Tables S2–S3). LG cases were not included due to low number of patients, reflecting the rarity of this disease, and because of their distinct disease characteristics as compared with HG cases. A threshold value could not be obtained by ROC curves analyses, and to avoid bias in selecting a cut-off for each protein, all staining scores (grouped as shown in Figure 1) were taken into account in deriving the Kaplan-Meier curves for each protein.

Increasing levels of cytoplasmic RAN staining in HG serous EOC was significantly associated with a poorer overall survival (p = 0.016; Figure 3A, Table S3). These observations are consistent with our initial findings [13]. Of the RAN network partners, the high cytoplasmic expression of XPO7 (p = 0.02; Figure 3E) and high nuclear TPX2 expression (p = 0.002, Figure 3I) were correlated with overall shorter survival.

To further investigate the potential prognostic significance of RAN and its partner proteins in overall patient survival, univariate Cox regression analyses were performed (Table 2). Cytoplasmic RAN and XPO7 staining and nuclear TPX2 localization in HG serous EOC were significantly correlated with poor overall survival (p = 0.008, p = 0.027, p = 0.002, respectively). These proteins were substantial independent prognostic factors for survival with hazard ratios (HR = 1.82 for RAN, HR = 1.58 for XPO7 and HR = 2.66 for TPX2) comparable to that of residual disease (HR = 1.82), a known prognostic factor [3].

**Table 2:** Univariate and multivariate Cox regression analyses. Statistical association between the expression of RAN or its partners and HG serous patient outcomes.

	Univariate Cox regression	
Biomarkers	Overall Survival*	Disease Free Survival**
RAN	**p = 0.008**	**p = 0.006**
	**HR = 1.825**	**HR = 1.698**
RANBP1	p = 0.618	p = 0.567
RCC1	p = 0.437	p = 0.166
IMPORTIN β	p = 0.461	p = 0.348
XPO7 cytoplasmic	**p = 0.027**	p = 0.339
	**HR = 1.584**	
XPO7 nuclear	p = 0.183	p = 0.883
XPOT cytoplasmic	p = 0.638	p = 0.656
XPOT nuclear	p = 0.515	p = 0.710
TPX2	**p = 0.002**	**p = 0.006**
	**HR = 2.652**	**HR = 2.560**
Residual Disease	**p<0.0001**	**p = 0.05**
	**HR = 1.825**	**HR = 1.281**
	Multivariate Cox regression	
Biomarkers	Overall Survival	Disease Free Survival
RAN	**p = 0.015**	**p = 0.013**
	**HR = 1.839**	**HR = 1.689**
Stage	p = 0.685	p = 0.625
Residual disease	**p = 0.003**	p = 0.219
	**HR = 1.617**	
TPX2	**p = 0.033**	**p = 0.024**
	**HR = 2.156**	**HR = 2.527**
Stage	p = 0.297	p = 0.705
Residual disease	**p = 0.035**	p = 0.607
	**HR = 1.449**	
XPO7 Cytoplasmic	p = 0.182	
Stage	p = 0.283	
Residual disease	**p = 0.002**	
	**HR = 1.501**	

*Overall survival is the time from the date of primary resection until either death due to ovarian cancer or last follow-up.

**Disease free survival is the time from the first resection of the primary tumor until the first event of recurrence.

Residual disease = amount of residual disease at time of primary resection of the ovarian tumor.

p = p-value. HR = hazard ratio. Bold fonts denote significant values.

Taken together, our results suggest that XPO7, involved in RAN’s nucleo-cytoplasmic export of proteins, and TPX2, a partner of RAN in mitosis, as well as RAN itself, may contribute to the malignant potential of serous EOC and influence patient’s overall survival.

### Association of RAN and TPX2 with Recurrence in Serous EOC Patients

**Figure 4:**
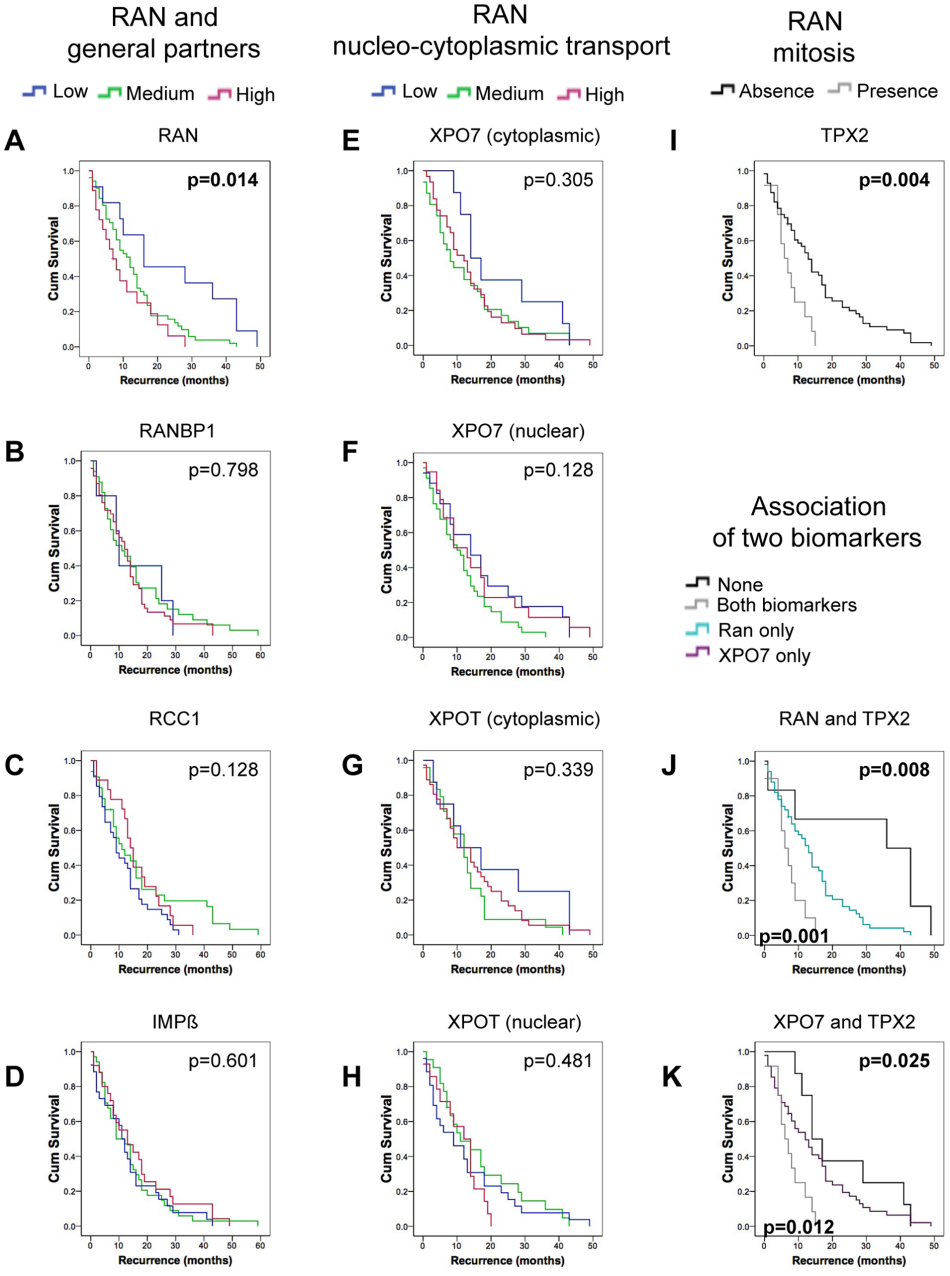
Ran and TPX2 expressions are associated with a short progression free survival in high-grade serous carcinoma. Evaluation of RAN network protein expression in only high-grade serous carcinomas by Kaplan–Meier analysis. Progression free survival corresponds to the time in months calculated from the time of first surgery until the first progression. The log-rank test was used to define statistical difference between groups. (See Figure 3 legend for details).

The importance of RAN network proteins in recurrence in the context of disease free survival was assessed by Kaplan-Meier curve and Cox regression analyses as described above. Increasing levels of cytoplasmic RAN staining in HG serous EOCs was significantly associated with shorter disease free survival by Kaplan-Meier analysis (p = 0.014; Figure 4A, Table S3). Of the RAN network proteins, Kaplan-Meier curve analyses found only nuclear TPX2 localization as significantly associated with recurrence in HG serous EOC (p = 0.004; Figure 4I, Table S3). Univariate Cox regression analyses validated these findings where RAN (p = 0.006) and TPX2 (p = 0.006) proteins showed significant correlation with disease recurrence (Table 2). These findings were also comparable to hazard ratios determined for residual disease (Table 2).

Therefore, our results suggest that RAN’s mitotic function specifically, via its TPX2 partner, appears to be associated with recurrence of HG serous EOC.

### Multivariate Analyses Confirms the Strong Association of TPX2 Expression in Patient Survival and Disease Recurrence

In order to further define the potential prognostic value of the RAN protein network, multivariate Cox regression analyses were performed for RAN and each partner that achieved significance in univariate analysis.

Hazard ratios of RAN, XPO7 and TPX2 for overall patient survival were compared to standard prognostic factors such as stage and residual disease (Table 2). Our results show that only RAN (p = 0.015, HR = 1.84), TPX2 (p = 0.033, HR = 2.15) and residual disease (p<0.05) were significant independent prognostic markers for overall survival in HG tumors. Interestingly, both RAN and TPX2 had higher HRs than residual disease (Table 2).

For recurrence, the pattern of expression of RAN and TPX2 were also compared to standard prognosis variables (stage and residual disease). In HG tumors, only RAN (p = 0.013, HR = 1.69) and TPX2 (p = 0.024, HR = 2.53) were independent variables predicting a high risk of recurrence (Table 2).

### A Concomitant Over-expression Pattern of TPX2/RAN or TPX2/XPO7 is Associated with a Poorer Outcome in Serous EOC Patients

Our results indicate that the expression patterns of RAN and its mitotic partner TPX2 in serous EOC act as independent prognostic biomarkers for both overall patient survival and recurrence. We next evaluated the combined impact of these two candidate biomarkers on overall and disease free survival using Kaplan-Meier curve analysis. Our results showed a significant difference between staining patterns of RAN and TPX2 in progression free survival (Figure 4J) in HG cases. In particular, the concomitant presence of RAN and TPX2 was associated with shorter disease free survival as compared to the presence of RAN alone (15 months versus 30 months, p = 0.001, Figure 4J). The same trend was also observed for overall patient survival with borderline significance (p = 0.07, Figure 3J). Using the same analyses, we also demonstrated that XPO7 and TPX2 expression patterns were significantly associated with both poorer overall survival (p = 0.012) and shorter disease free survival (p = 0.012) when compared to the presence of only XPO7 (Figure 3K, Figure 4K). It is notable that TPX2 expression, when it occurred, was always associated with either RAN or XPO7 co-expression.

## Discussion

High RAN expression levels have been reported in different tumors, such as pancreas, colon, lung, nasopharynx, stomach and kidney, when compared to normal tissues [17]–[19], [36]. Interestingly, RNA interference studies have shown that RAN down regulation drastically affects cancer cell survival but not that of normal cells [14], [18]. However, despite the vital importance of RAN in cancer cells it has been difficult to determine which function of this protein is involved in tumorigenesis. To begin to address this issue we have studied the expression of several RAN partners, specific to its distinct functions, in association with EOC patient survival and recurrence.

Using a larger cohort, we have corroborated our initial findings that patients with high RAN expression in serous EOC are associated with disease progression and poor outcome [12], [13]. Herein we demonstrated that cytoplasmic RAN expression was significantly higher in HG serous EOC than LG (Figure 2) and that this expression pattern has a predictive value for poor overall survival and a high risk of recurrence (Figures 3–4). However, to our knowledge, our group is the first to have conducted a systematic study that investigates the expression of RAN concomitant with its specific network partners. Higher expression level of several of RAN’s partners are noted in HG tumors compared to LG (Figure 2), in particular importin β and the proteins involved in the GDP-GTP cycle (RanBP1 and RCC1), which are implicated in both functions of RAN. In addition, the expression of these proteins correlated positively with that of RAN itself (Table 2). On the other hand, only partners of RAN involved in more specific roles, such as the nucleo-cytoplasmic transport (XPO7) and mitosis (TPX2), showed significant association with poor patient outcome in HG serous EOC (Figures 3–4). These findings suggest that a deregulation of RAN in HG tumors is accompanied by a deregulation in its general partners, to ensure its overall function. Thus as they are not associated with clinical outcomes it is perhaps the specific functions of RAN that ultimately drive tumor malignancy, cell transformation and contribute to poor patient outcome. We found that the expression of nuclear-cytoplasmic receptor for protein export XPO7 and the spindle assembly protein TPX2, are tightly linked to clinical outcomes suggesting a critical role for these specific functions in EOC.

The exportin XPO7 has only recently been described [30], [31] and is a member of the superfamily of β-related nuclear transport importin/exportin receptors [32], [33]. Though the functions of this exportin is unknown, XPO7 has recently been shown to exhibit a broad substrate specificity recognizing proteins that contain positively charged regions and to a lesser extent the classical NES (Nuclear Export Signal) [35], [37], [38]. These proteins include 14-3-3 (known to anchor cyclin-dependent kinases), p50RhoGAP (a GTPase-activating protein for Rho- and Rac-GTPases), STRAD α (a regulator of the serine/threonine kinase LKB1), and E2A (transcription factors of the basic-loop-helix family). Except for the latter, the biological activity of these proteins is dependent on their cytoplasmic localization, highlighting the importance of XPO7 on their role [35], [37], [38]. Our results showed that cytoplasmic localization of XPO7 predicts for poor patient survival and that nuclear localization of this exportin was decreased in HG serous EOC cases (Figures 2–3). This is the first study showing a clinical implication of this exportin in cancer. It will be interesting in the future to determine which of XPO7 cargoes are mostly associated with tumor malignancy and clinical progression. Furthermore, even limited expression of XPO7 was associated with poor overall patient survival (Figure 3) indicating that this protein could be a competent candidate biomarker for prognosis.

Apart from XPO7, we also found an association between high TPX2 staining and poor patient outcome in HG serous EOC (Figures 3–4). TPX2 is a mitotic spindle assembly factor, which is inhibited by the association with an importin. RAN-GTP causes the dissociation of the TPX2/importin inhibitory complex and this is spatially regulated [39], [40] by an established RAN-GTP/RAN-GDP gradient in proximity to the chromosome [35], [41]. As expected, TPX2 expression is always nuclear (Figure 1), and was exclusively observed in HG tumors (Figure 2). The role of TPX2 in cancer progression has been well characterized [39], [40], [42], [43]. Indeed, TPX2 has been associated with various cancers such as breast, lung, prostate, lymphoma and malignant astrocytoma, and has also been correlated with genomic instability [39], [40], [42], [43]. TPX2 gene is localized on chromosomal band 20q11, a region of frequent amplification with a strong correlation between copy number and protein expression levels [44], [45]. In addition, the important contribution of RAN to the mitotic process has also been well described [46]. Here, we report that TPX2 expression, like RAN, correlates positively with tumor grade, and was associated with poor patient survival and with a short recurrence time in HG serous EOC (Figures 2–4). Indeed, TPX2 provides information on HG serous EOC aggressiveness and could be an indicator of early recurrence. TPX2 and Aurora-A are genes differentially expressed in ovarian cancer [47]. In addition, these two proteins function to regulate the attachment of microtubules to the kinetochore during prophase, and aberrant expression of these proteins leads to aneuploidy and is speculated to contribute to cancer progression [48]. Inhibitors of Aurora-A, when administered along with taxane, lead to better overall survival in patients with taxane resistant ovarian cancer [47]. Combined with our present results, we speculate that deregulation of RAN’s mitotic function, via TPX2 protein, can lead to genetic instability and alterations, which are commonly observed in HG serous EOC. Furthermore, it is possible that targeting RAN and/or TPX2 may improve treatment response and decrease recurrence rate of serous EOC. Moreover, multivariate logistic-regression analysis revealed that RAN and TPX2 expression continued to be prognostic factors even after considering the effects of stage and residual disease. These results suggest that higher RAN or TPX2 expression can identify patients with poor prognosis (shorter overall and disease-free survival) even in the context of no residual disease.

Overall, our findings indicated that the RAN network, including both of RAN’s main functions (nucleo-cytoplasmic transport and mitosis), are deregulated in serous ovarian carcinomas affecting tumor progression and patient survival. However, multivariate analyses demonstrate a higher risk for patients expressing either RAN or TPX2 than for those expressing XPO7 (Table 2), suggesting a more significant contribution of RAN’s mitotic function to ovarian cancer progression than its nucleo-cytoplasmic function. Nevertheless, our results also indicated that a synergy between these functions might exist since concomitant expression of TPX2 and RAN or XPO7 had a more significant impact in HG serous EOC patient survival and recurrence than the expression of each of these latter proteins alone (Figure 3–4). Molecular studies have shown that down-regulation of RAN results in mitotic defects, the improper localization of TPX2, apoptosis and tumor growth inhibition [14], [18]. On the other hand, ectopic expression of RAN increases survival signals without cell cycle defects, inducing cell transformation, anchorage independent growth, invasion and metastasis [16], [49], [50]. We herein extend these *in vitro* studies showing that RAN associated nuclear export and mitotic spindle assembly correlate with clinical outcomes in serous ovarian carcinomas. Further dissection of the role of RAN, and its cellular functions, will need to be undertaken to fully understand their role in ovarian cancer.

## Supporting Information

Figure S1 **Specificity of antibodies by immunoblotting.** Western-blot analysis of whole cell lysate from four epithelial ovarian cancer cell lines (TOV81D, TOV2223G, TOV1946, TOV112D)*. Extracts were loaded on 8% or 10% SDS/PAGE gel and membranes were hybridized with anti-RAN (A), anti-RANBP1 (B), anti- RCC1 (C), anti-IMPβ (D), anti-XPOT (E), anti-XPO7 (F) and anti-TPX2 (G). Immunoblots were performed on four different proteins extracts from cell lines and representative images are presented. β-actin was used as a loading control. *V. Ouellet *et al.*, BMC Cancer. 2008. D. Provencher *et al*. In Vitro Cell Dev Biol Anim. 2000.
(TIF)

Figure S2 **Specificity of antibodies for RAN protein network on cell pellets.** Immunohistochemistry analyses were performed on paraffin-embedded cell pellets* of the four epithelial ovarian cancer cell lines (TOV81D, TOV2223G, TOV1946, TOV112D). Images are representative staining patterns for each member of RAN network corresponding to low (left panel) or high (right panel) expression (magnification 20 X). Note that high and low expression correlated between Western blot and immunohistochemistry on cell pellets. * Zietarska M *et al.*, Histopathology. 2010.
(TIF)

Table S1 Clinical parameters of the serous EOC samples in the cohort.
(DOCX)

Table S2 Number of HG serous EOC patients for each RAN partner in every Kaplan-Meier curve.
(DOCX)

Table S3 Kaplan-Meier analysis of RAN network in HG serous EOC.
(DOCX)
