# Supplementary figures and images for: RAN Nucleo-Cytoplasmic Transport and Mitotic Spindle Assembly Partners XPO7 and TPX2 Are New Prognostic Biomarkers in Serous Epithelial Ovarian Cancer
Source: PLoS One. 2014 Mar 13;9(3):e91000. doi: 10.1371/journal.pone.0091000 (PMC3953127; doi:10.1371/journal.pone.0091000)

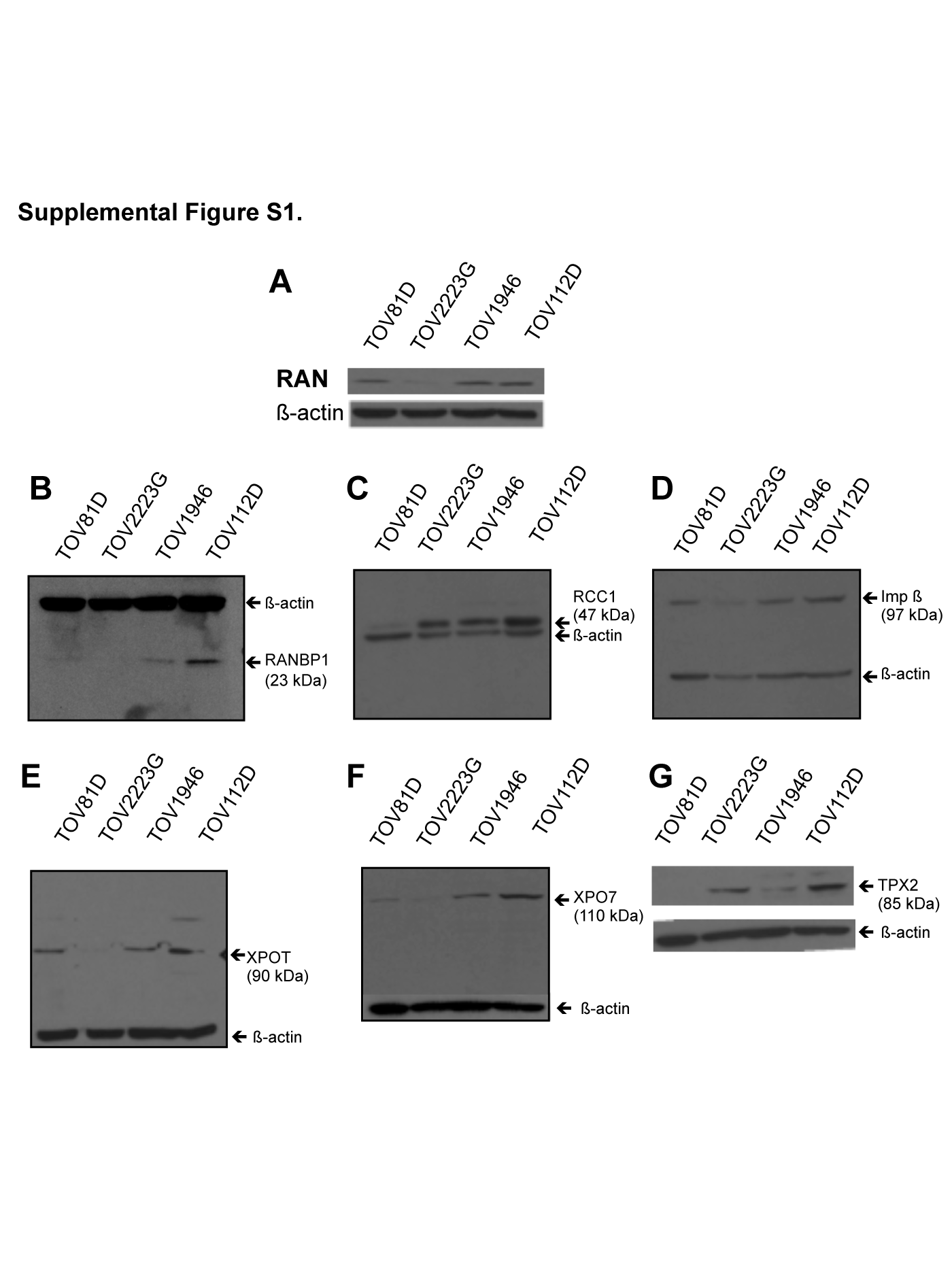

Supplement: Figure S1 — Specificity of antibodies by immunoblotting. Western-blot analysis of whole cell lysate from four epithelial ovarian cancer cell lines (TOV81D, TOV2223G, TOV1946, TOV112D)*. Extracts were loaded on 8% or 10% SDS/PAGE gel and membranes were hybridized with anti-RAN (A), anti-RANBP1 (B), anti- RCC1 (C), anti-IMPβ (D), anti-XPOT (E), anti-XPO7 (F) and anti-TPX2 (G). Immunoblots were performed on four different proteins extracts from cell lines and representative images are presented. β-actin was used as a loading control. *V. Ouellet et al., BMC Cancer. 2008. D. Provencher et al. In Vitro Cell Dev Biol Anim. 2000. (TIF) [file pone.0091000.s001.tif]

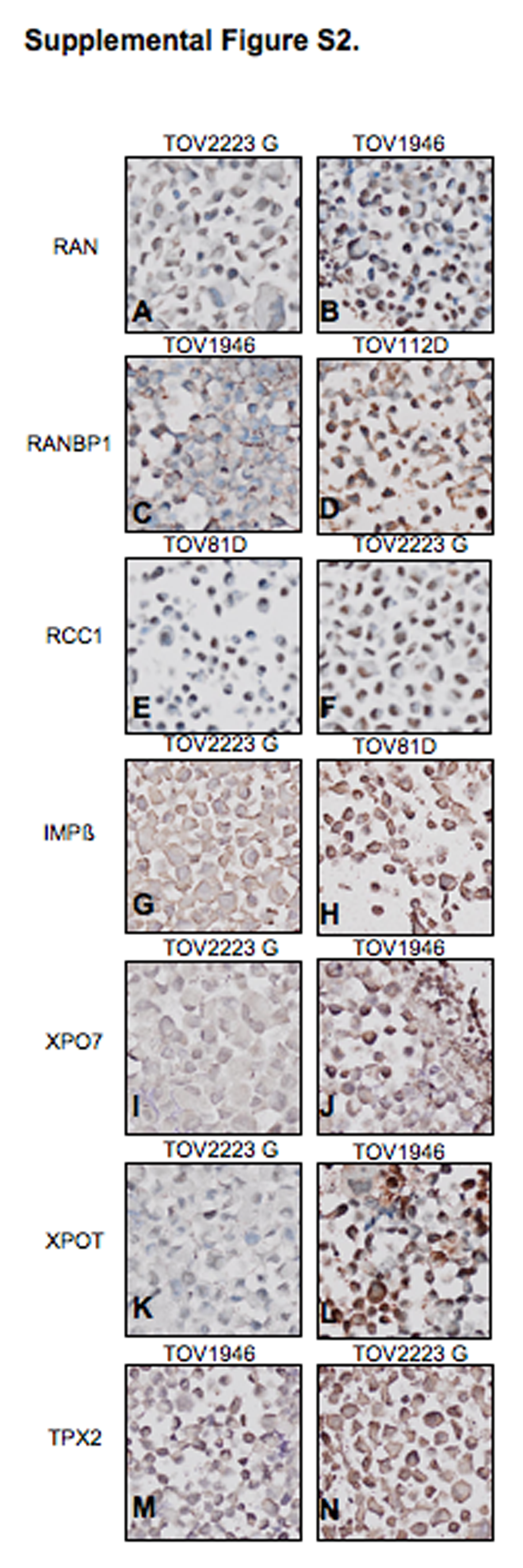

Supplement: Figure S2 — Specificity of antibodies for RAN protein network on cell pellets. Immunohistochemistry analyses were performed on paraffin-embedded cell pellets* of the four epithelial ovarian cancer cell lines (TOV81D, TOV2223G, TOV1946, TOV112D). Images are representative staining patterns for each member of RAN network corresponding to low (left panel) or high (right panel) expression (magnification 20 X). Note that high and low expression correlated between Western blot and immunohistochemistry on cell pellets. * Zietarska M et al., Histopathology. 2010. (TIF) [file pone.0091000.s002.tif]
