# Supplementary material for: RAN Nucleo-Cytoplasmic Transport and Mitotic Spindle Assembly Partners XPO7 and TPX2 Are New Prognostic Biomarkers in Serous Epithelial Ovarian Cancer
Source: PLoS One. 2014 Mar 13;9(3):e91000. doi: 10.1371/journal.pone.0091000 (PMC3953127; doi:10.1371/journal.pone.0091000)
Supplement: Table S1 — Clinical parameters of the serous EOC samples in the cohort. (DOCX) [file pone.0091000.s003.docx]

**Table S1.** Clinical parameters of the serous EOC samples in the cohort

| Serous EOC |  | LG* | HG* |
| --- | --- | --- | --- |
|  |  | (n=12) | (n=131) |
| Stage** (number of patients) | I | 0 | 4 |
|  | II | 0 | 8 |
|  | III | 12 | 102 |
|  | IV | 0 | 15 |
|  | Missing | 0 | 2 |
| Average age of patients (years) |  | 48.5 (27-71) | 62.6 (34-87) |
| Average follow-up (months)*** |  | 50.6 (5-205) | 37.2 (1-142) |
| Average recurrence (months)*** |  | 16.7 (1-53) | 14.2 (1-59) |

####

*Histological grade of the tumor was determined by a pathologist and divided in two groups: low grade (LG) and high grade (HG).

**Stage was separated in four categories according to the FIGO classification.

***Follow up and recurrence is the time in months from the date of primary resection of the ovarian tumor until death due to ovarian cancer or last contact with the patient while recurrence is defined from initial surgery until the first event of disease progression.
