# Supplementary material for: RAN Nucleo-Cytoplasmic Transport and Mitotic Spindle Assembly Partners XPO7 and TPX2 Are New Prognostic Biomarkers in Serous Epithelial Ovarian Cancer
Source: PLoS One. 2014 Mar 13;9(3):e91000. doi: 10.1371/journal.pone.0091000 (PMC3953127; doi:10.1371/journal.pone.0091000)
Supplement: Table S2 — Number of HG serous EOC patients for each RAN partner in every Kaplan-Meier curve. (DOCX) [file pone.0091000.s004.docx]

**Table S2.** Number of HG serous EOC patients for each RAN partner in every Kaplan-Meier curve.

| **Biomarker** | **Category** | **Overall survival** | **Disease free survival** |
| --- | --- | --- | --- |
| RAN | <1 | 17 | 11 |
|  | =1<2 | 76 | 51 |
|  | ≥2 | 27 | 18 |
| RANBP1 | <1 | 5 | 5 |
|  | =1<2 | 47 | 33 |
|  | ≥2 | 76 | 46 |
| RCC1 | <1 | 53 | 34 |
|  | =1<2 | 47 | 32 |
|  | ≥2 | 28 | 18 |
| IMPß | <1 | 36 | 26 |
|  | =1<2 | 57 | 34 |
|  | ≥2 | 36 | 25 |
| XPO7 cytoplasmic | <1 | 14 | 8 |
|  | =1<2 | 49 | 31 |
|  | ≥2 | 44 | 31 |
| XPO7 nuclear | <1 | 34 | 17 |
|  | =1<2 | 51 | 34 |
|  | ≥2 | 22 | 19 |
| XPOT cytoplasmic | <1 | 11 | 8 |
|  | =1<2 | 39 | 24 |
|  | ≥2 | 54 | 36 |
| XPOT nuclear | <1 | 38 | 26 |
|  | =1<2 | 37 | 22 |
|  | ≥2 | 18 | 14 |
| TPX2 | 0 | 86 | 56 |
|  | >0 | 19 | 12 |
| RAN+TPX2 | none | 15 | 10 |
|  | RAN | 9 | 6 |
|  | RAN+TPX2 | 75 | 50 |
| XPO7+TPX2 | none | 14 | 8 |
|  | XPO7 | 72 | 48 |
|  | XPO7+TPX2 | 19 | 12 |
