# Supplementary material for: RAN Nucleo-Cytoplasmic Transport and Mitotic Spindle Assembly Partners XPO7 and TPX2 Are New Prognostic Biomarkers in Serous Epithelial Ovarian Cancer
Source: PLoS One. 2014 Mar 13;9(3):e91000. doi: 10.1371/journal.pone.0091000 (PMC3953127; doi:10.1371/journal.pone.0091000)
Supplement: Table S3 — Kaplan-Meier analysis of RAN network in HG serous EOC. (DOCX) [file pone.0091000.s005.docx]

**Table S3.** **Kaplan-Meier analysis of RAN network in HG serous EOC**

|  | Biomarkers | Overall | Disease Free |
| --- | --- | --- | --- |
|  |  | survival* | survival**^**^** |
| RAN and | RAN | **p=0.016** | **p=0.014** |
| general |  | **X^2^=8.217** | **X^2^=8.572** |
| partners | RANBP1 | p=0.872 | p=0.798 |
|  | RCC1 | p=0.690 | p=0.128 |
|  | IMPORTIN ß | p=0.610 | p=0.601 |
| Nucleo- | XPO7 | **p=0.02** | p=0.305 |
| cytoplasmic | cytoplasmic | **X^2^=7.781** |  |
| transport | XPO7 | p=0.150 | p=0.128 |
|  | nuclear |  |  |
|  | XPOT | p=0.244 | p=0.339 |
|  | cytoplasmic |  |  |
|  | XPOT | p=0.797 | p=0.481 |
|  | nuclear |  |  |
| Mitosis | TPX2 | **p=0.002** | **p=0.004** |
|  |  | **X^2^=10.049** | **X^2^=8.393** |

*Overall survival is the time from the date of primary resection until either death due to ovarian cancer or last follow-up.

**Disease free survival is the time from the first resection of the primary tumor until the first event of recurrence.

X^2^ = log rank; p= p-value. Bold fonts denote significant values.
